# Prenatal THC Exposure Differentially Alters Gut Physiology and Microbiota in Stress-Resilient and Vulnerable Mice

**DOI:** 10.3390/ph19091463

**Published:** 2026-09-15

**Authors:** Mohamed Mari, Oshrit Rahimi, Ronaldo de A. A. Junior, Beatriz G. S. Rocha, Anastasia Bagaev, Debpali Sur, Dilorom Begmatova, Natalia Zemliana, Andy Boiangiu, Panayotis K. Thanos, Kenneth Blum, Natalya M. Kogan, Albert Pinhasov

**Affiliations:** 1 Institute of Personalized and Translational Medicine, Ariel University, Ariel 40700, Israel; mohamedm@ariel.ac.il (M.M.); rjunior.psi@gmail.com (R.d.A.A.J.); beatriz.gsrocha@gmail.com (B.G.S.R.); anastasiaba@ariel.ac.il (A.B.); debpali.rony@gmail.com (D.S.); dilorom0508@gmail.com (D.B.); nataliaz@ariel.ac.il (N.Z.); andyb@013.net (A.B.); thanos@buffalo.edu (P.K.T.); drd2gene@gmail.com (K.B.); 2 School of Biomedicine, Adelson Faculty of Medicine, Ariel University, Ariel 40700, Israel; oshritra@ariel.ac.il; 3 Behavioral Neuropharmacology and Neuroimaging Laboratory on Addictions, Clinical Research Institute on Addictions, Department of Pharmacology and Toxicology, Jacobs School of Medicine and Biosciences, State University of New York at Buffalo, Buffalo, NY 14203, USA; 4 Division of Addiction Research & Education, Center for Sports, Exercise, Psychiatry, Western University Health Sciences, Pomona, CA 91766, USA

**Keywords:** THC, cannabis, microbiome, dominance, submissiveness, stress resilience, stress-coping abilities, social behavior, GBA, gut, *CB1R*

## Abstract

**Background/Objectives:** Prenatal environmental exposures can shape neurodevelopmental outcomes through the gut–brain axis. The endocannabinoid system contributes to both neurodevelopment and gastrointestinal homeostasis and may, therefore, be vulnerable to disruption by exogenous cannabinoids such as Δ^9^-tetrahydrocannabinol (THC). Using established dominant (Dom; stress-resilient) and submissive (Sub; stress-vulnerable) mouse lines, this study examined whether prenatal THC exposure (PTE) produces phenotype-dependent alterations in gut physiology, colonic endocannabinoid- and inflammation-related gene expression, and gut microbiota composition. **Methods:** Pregnant Dom and Sub mice received vehicle or THC during gestation. Maternal stool samples were analyzed after exposure, and offspring were assessed at postnatal day 30. Gut and colon lengths were measured; colonic mRNA expression of selected endocannabinoid-, barrier-, and inflammation-related genes was quantified by qRT-PCR; and gut microbiota composition was analyzed using 16S rRNA sequencing with diversity and differential-abundance analyses. **Results:** PTE induced phenotype-dependent alterations in maternal gut microbiota. Sub dams exhibited enrichment of taxa associated with inflammatory states and depletion of Bacteroides and Parabacteroides, whereas Dom dams showed no significant taxonomic shifts. In offspring, Sub mice showed shorter baseline gut and colon lengths than Dom mice. PTE reduced gut and colon length in Dom offspring but not in Sub offspring. PTE also increased colonic *CB1R* expression in Dom offspring and reduced *PPARγ* expression in Sub offspring. Microbiota analysis revealed bidirectional phenotype-dependent remodeling, including increased Lachnospiraceae-related taxa and reduced Ligilactobacillus in Dom offspring, with an opposite pattern in Sub offspring. **Conclusions:** PTE produces phenotype-dependent effects on gut physiology, colonic gene expression, and microbiota composition. These findings identify the host stress-coping phenotype as a potential modifier of developmental responses to prenatal THC but do not establish causal relationships among microbiota, intestinal outcomes, and previously reported behavioral effects.

## 1. Introduction

With cannabis gaining broader social acceptance, its usage rates have climbed steadily, including among pregnant women [1]. Cannabinoids interact with the endocannabinoid system (ECS), which is essential for fetal development [2], including neural [3], immune [4], and gastrointestinal (GI) system maturation [5]. Exogenous cannabinoids such as Δ^9^-tetrahydrocannabinol (THC) act through components of the same cannabinoid receptor signaling system that regulates fetal development and gastrointestinal homeostasis. Prenatal THC exposure may perturb both neurodevelopmental and gut developmental trajectories [3,5].

Gut microbiota are critical for maintaining homeostasis [6] and for shaping brain functions [7,8]. These effects are mediated through the bidirectional cross-talk of the gut–brain axis, involving metabolic [9], nutritional [10], endocrine [11], and immunological aspects [11]. Disruptions of gut morphology and microbiota composition during critical developmental periods can have lasting physiological and behavioral consequences [12]. The ECS regulates gut permeability, motility, and microbial composition, and alterations in its signaling during gestation lead to long-term GI and metabolic dysfunctions [13]. Several studies have demonstrated that prenatal cannabis exposure can alter gut morphology and microbial diversity, inflammatory responses, and metabolic profiles, which may contribute to systemic effects observed in adulthood [14,15,16,17,18].

Due to high heterogeneity in the human population, different environmental factors may influence the extent of cannabis effects on prenatal or postnatal stages of development [19,20]. Stress is one of the factors, and the ability of organisms to cope with stressogenic situations may largely dictate developmental changes and responses [21]. We have previously described a mouse model developed by us of social dominance (Dom) and submissiveness (Sub) that exhibit inherited stress resilience and vulnerability, respectively [22,23,24,25,26,27]. We have shown that Dom and Sub mice exhibited differential responses to psychotropic agents [24], differences in brain neurochemical profiles [23], and a distinct immune profile [28]. Our recent findings revealed that Dom and Sub mice possess distinct gut microbiota compositions, with Sub mice exhibiting shorter gut and colon lengths. Furthermore, microbiota-transfer experiments using Dom- and Sub-derived microbiota demonstrated that gut microbiota contribute to line-associated social and physiological phenotypes, supporting the relevance of microbiome analysis in the present PTE study.

Using these previously characterized stress-resilient and stress-vulnerable phenotypes, the present study evaluated whether PTE-induced phenotype-dependent behavioral outcomes are paralleled by alterations in maternal and offspring gut microbiota, offspring intestinal morphology, and colonic expression of genes related to endocannabinoid signaling and inflammatory regulation.

## 2. Results

### 2.1. Dams

#### Behavioral Phenotype Is Associated with Dams’ Gut Microbiota Composition with PTE-Associated Shifts in Sub Dams

**Figure 1:**
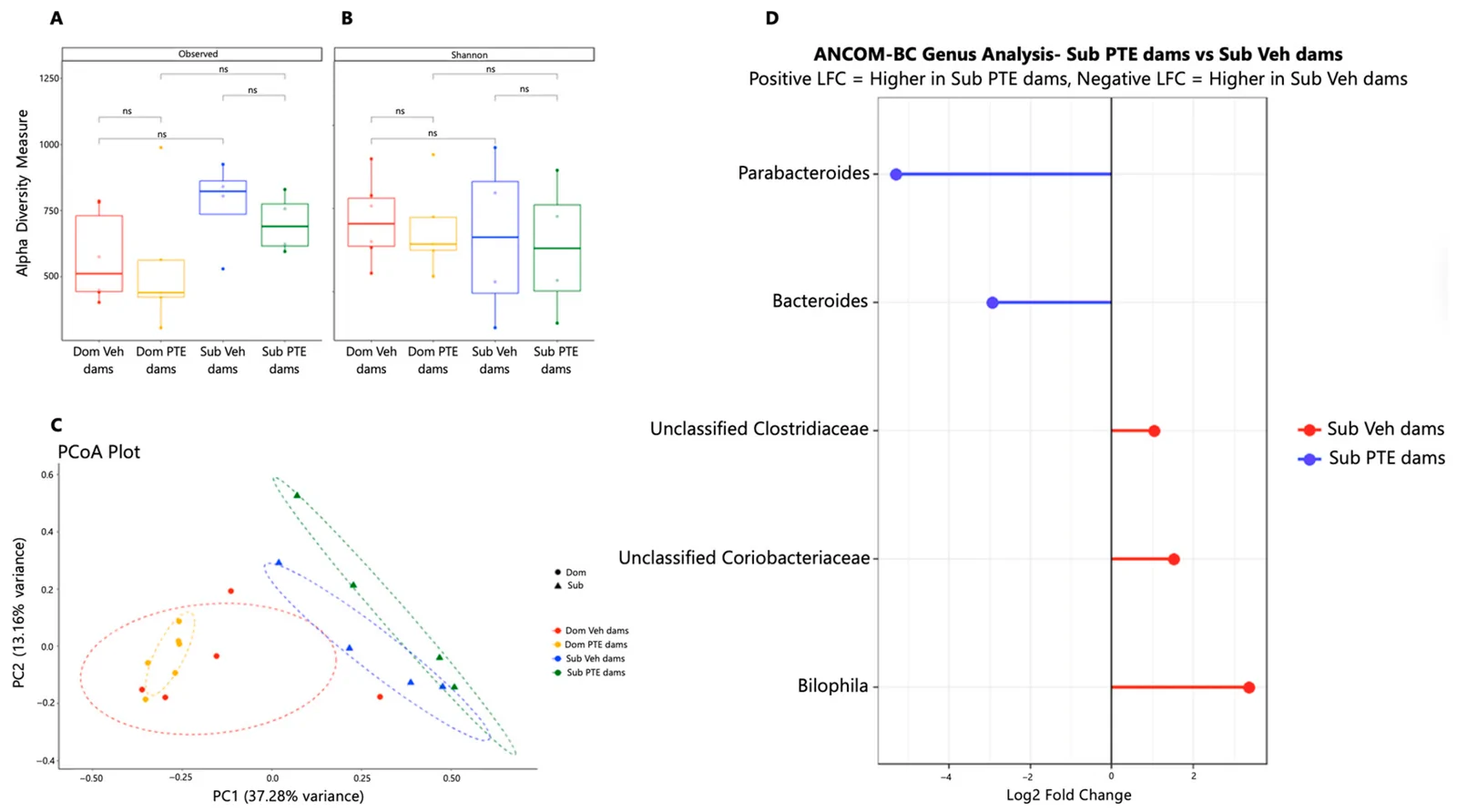
Dams’ gut microbiota composition following PTE. (**A**,**B**) α diversity showed no significant differences among Dom Veh, Dom PTE, Sub Veh, and Sub PTE dams. (**C**) PCoA based on Bray–Curtis dissimilarity demonstrates phenotype-dependent clustering (PC1 = 37.28% variance). (**D**) Differential abundance analysis (ANCOM-BC) identified enrichment of *Bilophila*, unclassified *Clostridiaceae*, and unclassified *Coriobacteriaceae* and depletion of *Parabacteroides* and Bacteroides in Sub PTE dams. (ns)—a non-significant difference; (**A**–**C**) Red, orange, blue, and green represent Dom Veh, Dom PTE, Sub Veh, and Sub PTE dams, respectively; (**D**) Red indicates Sub Veh dams and blue indicates Sub PTE dams. No significant taxa were detected in the Dom dams (*n* = 4–5/group).

Gut microbiota profiling was performed on stool samples collected from dams 24 h after the final THC injection (Figure 1A,B). The α-diversity metrics, including observed richness and Shannon diversity indices, showed no significant main effect of phenotype or PTE and no phenotype × treatment interaction among dams, indicating preserved species richness and evenness across groups.

In contrast, β-diversity analysis using Bray–Curtis dissimilarity revealed a clear separation by behavioral phenotype (Figure 1C), with Dom and Sub dams forming distinct clusters along PC1 (28% explained variance).

This separation was supported by PERMANOVA, revealed a significant phenotype effect (pseudo-F_1,15_ = 6.03, R^2^ = 0.262, *p* = 0.001, 999 permutations), whereas neither the PTE effect (pseudo-F_1,15_ = 0.97, R^2^ = 0.042, *p* = 0.393) nor the phenotype × PTE interaction (pseudo-F_1,15_ = 1.02, R^2^ = 0.044, *p* = 0.320) reached significance. Homogeneity of multivariate dispersion did not differ significantly among groups (betadisper, all *p* > 0.6), confirming that the phenotype effect reflects true compositional differences. Treatment effects were modest at the global level, and pairwise comparison did not detect significant separation between Sub PTE and Sub Veh dams (pseudo-F_1,6_ = 0.71, R^2^ = 0.105, *p* = 0.69). Despite the absence of a global compositional shift, genus-level differential abundance analysis using ANCOM-BC identified specific taxonomic shifts in Sub dams exposed to PTE (Figure 1D). Sub PTE dams showed enrichment of unclassified *Clostridiaceae* (q = 0.01767) and an unclassified genus within the family *Coriobacteriaceae* (q = 0.01767), as well as *Bilophila* (q = 0.01767). Conversely, *Parabacteroides* (q < 0.0001) and *Bacteroides* (q = 0.04706) were significantly reduced relative to Sub Veh controls. No significantly different taxa abundance among the Dom dams’ groups were detected.

### 2.2. Offspring

#### 2.2.1. PTE Affected the Offspring Gut and Colon Lengths in a Phenotype-Dependent Manner

**Figure 2:**
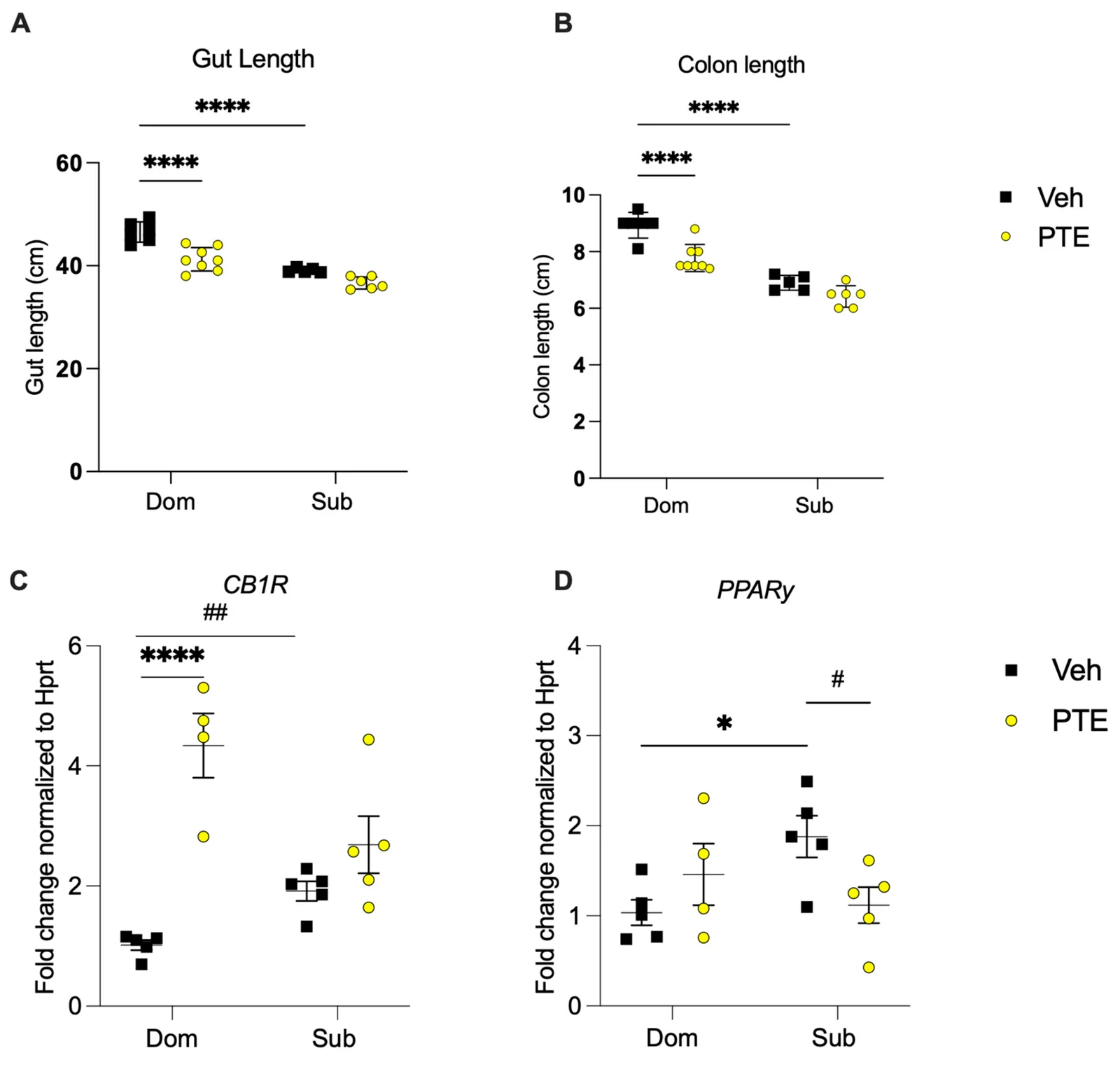
Effects of PTE on gut and colon length and colonic mRNA expression in Dom and Sub offspring (PND30). (**A**) Sub Veh offspring showed shorter gut length than Dom Veh. PTE significantly reduced gut length in Dom offspring compared with vehicle-treated counterparts. (**B**) Sub Veh displayed a shorter colon length than Dom Veh. PTE reduced colon length in Dom offspring with no effect in Sub. (**C**) *CB1R* mRNA expression was higher in Sub Veh compared with Dom Veh and significantly upregulated in Dom PTE compared with Dom Veh. (**D**) *PPARγ* expression was higher in Sub Veh than in Dom Veh. PTE decreased *PPARγ* in Sub offspring, with no effect in Dom. Data are mean ± SEM (gut/colon: *n* = 6–8/group, mRNA: *n* = 4–5/group). Statistical analysis was performed by two-way ANOVA (Sidak correction) for colon/gut lengths and mRNA expression; * at *p* < 0.05, and **** at *p* < 0.0001, or Student’s *t*-test with *p* value as # at *p* < 0.05, ## at *p* < 0.01.

We previously demonstrated that the stress vulnerability of Sub mice is associated with increased inflammation and shorter colon and gut lengths compared with Dom mice [29,30]. Prior to analysis, we verified the lack of dam-dependent effects by two-way ANOVA for each mouse strain by treatment (for colon lengths, Dom Veh F(1,2) = 0.1060, *p* = 0.7757; Dom THC F(2,3) = 0.9016, *p* = 0.4963; Sub Veh F(1,1) = 1, *p* = 0.5; Sub THC F(1,2) = 0.25, *p* = 0.6667 and for gut lengths, Dom Veh F(1,2) = 0.05512, *p* = 0.8362; Dom THC F(2,3) = 2.554, *p* = 0.2251; Sub Veh F(1,1) = 0, *p* > 0.9999; Sub THC F(1,2) = 0.005917, *p* = 0.9457). Consistent with these findings, two-way ANOVA showed a phenotype and treatment effect in gut (F_interaction_ (1,21)= 4.185, *p* = 0.0535; F_strain_ (1,21) = 72.65, *p* < 0.0001; F_treated_ (1,21) = 29.90, *p* < 0.0001) and colon lengths ((F_interaction_ (1,21) = 4.005, *p* = 0.0585; F_strain_ (1,21) = 101.5, *p* < 0.0001; F_treated_ (1,21) = 23.83, *p* < 0.0001)), with a significantly shorter gut length (*p* < 0.0001) and colon length (*p* < 0.0001) at PND 30 (Figure 2A,B). Surprisingly, PTE resulted in a significant reduction in gut (Figure 2A) and colon (Figure 2B) lengths in Dom offspring (*p* < 0.0001, *p* < 0.0001, respectively). However, no significant effect of PTE on gut or colon lengths was observed in the Sub offspring.

#### 2.2.2. PTE Altered Offspring Colonic *CB1R* and *PPARγ* mRNA Expression in a Phenotype-Dependent Manner

Prior to analysis, we verified the lack of dam-dependent effects by two-way ANOVA for each mouse strain by treatment (for mRNA *CB1R*, Dom Veh F(1,1) = 2.82, *p* = 0.3415; Dom PTE F(1,1) = 6.209, *p* = 0.2403; Sub Veh F(1,1) = 0.02721, *p* = 0.6939; Sub THC F(1,1) = 2.735, *p* = 0.3462; mRNA *PPARγ*, Dom Veh F(1,1) = 0.02226, *p* = 0.9057; Dom THC F(1,1) = 53.97, *p* = 0.0861; Sub Veh F(1,1) = 0.4676, *p* = 0.6182; Sub THC F(1,1) = 0.3817, *p* = 0.6477). To assess the long-term effects of PTE, colonic mRNA expression of genes related to endocannabinoid signaling and inflammatory regulation was analyzed in offspring at PND 30. Statistical analysis of colonic expression of mRNA *CB1R* (F_interaction_ (1,15) = 13.34, *p* = 0.0024; F_strain_ (1,15) = 1.159, *p* = 0.2987; F_treated_ (1,15) = 34.35, *p* < 0.0001). Under vehicle conditions, *CB1R* expression was higher in Sub compared with Dom offspring (t (7) = 6.937, *p* = 0.0002). Post hoc analysis indicated that PTE increased *CB1R* mRNA levels in Dom offspring relative to the Dom Veh group (*p* < 0.0001), whereas no significant difference was detected between the Sub PTE and Sub Veh groups. For colonic mRNA *PPARγ,* statistical analysis (F_interaction_ (1,15) = 6.759, *p* = 0.0201; F_strain_ (1,15) = 1.215, *p* = 0.2878; F_treated_ (1,15) = 0.5581, *p* = 0.4666) (Figure 2D), Sub Veh offspring exhibited higher expression compared with Dom Veh (*p* = 0.0328). PTE reduced *PPARγ* mRNA levels in Sub offspring relative to their vehicle controls (t (8) = 2.499, *p* = 0.037), while no significant change was observed in Dom offspring. No significant differences were detected in colonic *FAAH*, *CLDN4*, *CLDN7*, and *GPR43* mRNA expression between treated and vehicle groups (Supplementary Figure S2).

#### 2.2.3. PTE Reshapes Offspring Gut Microbiota Diversity and Taxonomic Composition in a Phenotype-Dependent Manner

**Figure 3:**
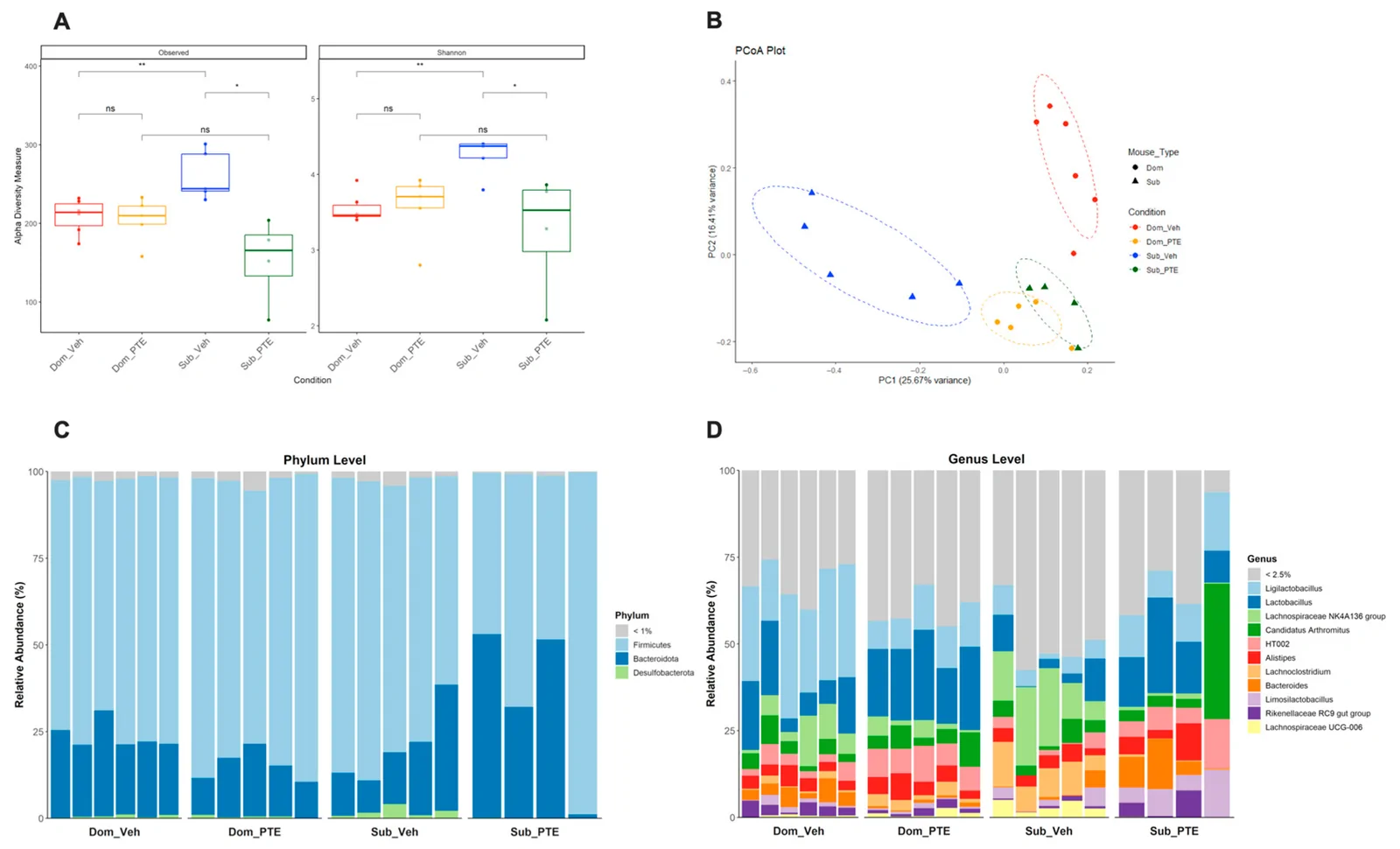
Effects of PTE on gut microbiota diversity and composition in Dom and Sub offspring. Gut microbial community diversity, structure, and composition across four experimental groups defined by social status (Dom or Sub) and exposure (Vehicle or PTE): Dom Veh (orange), Dom PTE (red), Sub Veh (blue), and Sub PTE (green). (**A**) α diversity (Shannon index and observed features). Sub Veh exhibited higher α diversity compared with the Sub PTE and Dom Veh groups. (**B**) Principal coordinates analysis (PCoA) based on weighted Bray–Curtis dissimilarity showing the β diversity across groups. Each point represents an individual sample; ellipses denote 70% confident intervals. PC1 and PC2 explain 25.67% and 16.41% of variance, respectively. (**C**,**D**) Relative abundance of bacterial taxa at the phylum (**C**) and genus (**D**) levels. Firmicutes and Bacteroidetes predominate across groups (>90% total abundance). PTE is associated with opposite directional shifts in Firmicutes and Bacteroidetes in the Dom versus Sub offspring. At the phylum level, taxa with median abundance > 1% are displayed individually; remaining taxa are grouped as “<1%”. At the genus level, taxa with median abundance > 2.5% are shown; others are grouped as “<2.5%”. Each bar represents one sample, with colors indicating taxonomic groups, *n* = 5/group. (ns)—a non-significant difference, (*) *p* < 0.05, and (**) *p* < 0.01.

We next explored whether PTE altered gut microbial diversity and composition in Dom and Sub offspring. At baseline, Sub Veh offspring exhibited higher α diversity compared with Dom Veh (Figure 3A). PTE was associated with a reduction in α diversity in Sub offspring, whereas no significant changes were detected in Dom offspring. β diversity analysis using Bray–Curtis dissimilarity demonstrated a clear separation between the Dom Veh and Sub Veh groups (Figure 3B, *p* = 0.004) consistent with previously reported strain-dependent microbial signatures [29]. PERMANOVA confirmed significant effects of phenotype (pseudo-F_1,16_ = 4.40, R^2^ = 0.152, *p* = 0.001), PTE (pseudo-F_1,16_ = 3.90, R^2^ = 0.135, *p* = 0.001), and a significant phenotype × PTE interaction (pseudo-F_1,16_ = 4.57, R^2^ = 0.158, *p* = 0.001; 999 permutations). Homogeneity of multivariate dispersion differed significantly by phenotype (betadisper, F_1,18_ = 9.57, *p* = 0.007) and across the four groups (F_3,16_ = 4.66, *p* = 0.004), but not by PTE exposure alone (*p* = 0.168), a factor to consider when interpreting the magnitude of the phenotype effect. PTE shifted microbial community structure in both phenotypes, resulting in four distinct clusters. Furthermore, Dom PTE and Sub PTE profiles exhibited increased similarity relative to their vehicle-treated counterparts (Figure 3B, *p* = 0.001, *p* = 0.0019, respectively). Taxonomic profiling revealed clear differences in microbial composition across groups (Figure 3C,D). At the phylum level (Figure 3C), *Firmicutes* and *Bacteroidetes* together comprised more than 90% of the microbial community in all samples. In the Dom offspring, PTE was associated with an increased relative abundance of *Firmicutes* (82.15 ± 8.16) relative to their vehicle control (Dom Veh: 74.18 ± 4.39), while the *Bacteroidetes* level was higher in Dom Veh (23.20 ± 4.04) compared with Dom PTE offspring (14.80 ± 4.46). In contrast, Sub PTE offspring revealed a reduction in *Firmicutes* (64.89 ± 24.49 vs. 76.84 ± 10.47 in Sub Veh) accompanied by an increase in *Bacteroidetes* (34.39 ± 24.15 vs. 18.87 ± 10.73 in Sub Veh). The relative bacterial abundance at the genus level among experimental groups is shown in Figure 3D.

#### 2.2.4. PTE Leads to Phenotype-Specific Shifts in Microbial Genera Abundance

**Figure 4:**
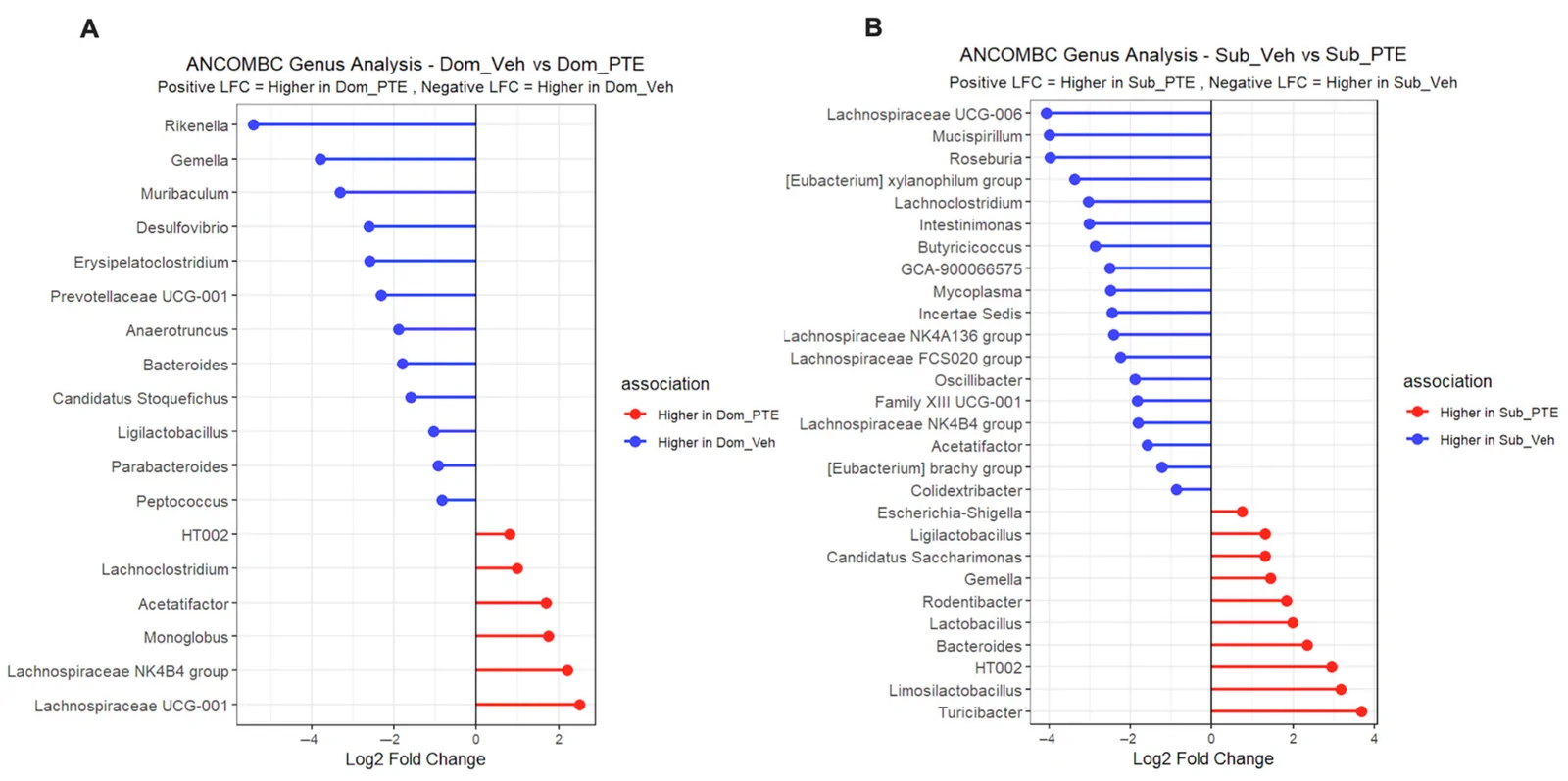
Differential gut microbiota composition in offspring following prenatal THC exposure. (**A**) Genus-level differential abundance analysis in Dom offspring (Dom Veh vs. Dom PTE) using ANCOMBC. Positive log2 fold change values (red) indicate genera significantly enriched in Dom PTE offspring, whereas negative values (blue) indicate genera enriched in Dom Veh offspring. (**B**) Genus-level differential abundance analysis in Sub offspring (Sub Veh vs. Sub PTE) using ANCOMBC. Positive log2 fold change values (red) indicate genera significantly enriched in Sub PTE offspring, whereas negative values (blue) indicate genera enriched in Sub Veh offspring. Only genera with statistically significant differences are shown. Statistical significance was assessed using the Wald test with Benjamini–Hochberg FDR correction (q-value < 0.05), *n* = 5/group.

Genus-level differential abundance was assessed using ANCOM-BC. In the Dom offspring (Figure 4A), 18 genera differed between Dom Veh and Dom PTE. The PTE resulted in a significant enrichment of six distinct taxa, including *Lachnospiraceae UCG-001* (q = 0.0002), the *Lachnospiraceae NK4B4* group (q < 0.0001), *Monoglobus* (q = 0.0234), *Acetatifactor* (q = 0.0038), *Lachnoclostridium* (q = 0.033), and *Lactobacillaceae HT002* (q = 0.0055). Conversely, 12 taxa were depleted in the Dom PTE group, representing a substantial reduction in their relative abundance (negative LFC), with the strongest reductions observed in *Rikenella, Gemella, Muribaculum, Desulfovibrio, Bacteroides,* and *Ligilactobacillus* (in all q < 0.0001) as well as *Erysipelatoclostridium, Prevotellaceae UCG-001, Candidatus Stoquefichus* (in all q < 0.01) and *Anaerotruncus, Parabacteroides,* and *Peptococcus* (q < 0.05). In contrast, in Sub offspring 28 genera were significantly altered, indicating a broader treatment-associated shift (Figure 4B). Ten taxa were enriched in Sub PTE (positive LFC): *Turicibacter* (q < 0.0001), *Saccharimonas* (q = 0.0003), *Lactobacillus* (q = 0.0099), *Candidatus Rodentibacter* (q = 0.0036), *Ligilactobacillus* (q = 0.0072), *Limosilactobacillus* (q = 0.0426), *Lactobacillaceae HT002* (q = 0.0472), *Bacteroides* (q = 0.0254), *Gemella* (q = 0.0204), and *Escherichia–Shigella* (q = 0.0418). Several taxa were reduced upon PTE administration, notably multiple *Lachnospiraceae* members (UCG-006, NK4A136 group, FCS020 group, q < 0.0001), as well as *Mucispirillum, Roseburia, Butyricicoccus xylanophilum* group, *Incertae Sedis*, *Oscillibacter*, *Lachnoclostridium*, *GCA-900066575* (all q < 0.001), *Intestinimonas, Mycoplasma, Family XIII UCG-001, Acetatifactor*, and *Colidextribacter* (all q = 0.03).

## 3. Discussion

This study investigated the consequences of prenatal THC exposure (PTE) on gut morphometry and microbial diversity in selectively bred dominant and submissive mouse phenotypes with distinct behavioral and stress coping abilities (Dom, stress-resilient and Sub, stress-vulnerable) [22,23,25]. Using this model, we demonstrated previously that PTE differentially affects behavioral outcomes, increasing anxiety in Dom offspring while improving sociability in Sub offspring, alongside differential expression of dopaminergic and endocannabinoid-related genes in the hippocampus and prefrontal cortex [31]. Here, we showed that the consequences of PTE during critical stages of neurodevelopment [32] produced phenotype-dependent effects on gut morphology, microbiota composition, and colonic gene expression, highlighting stress-coping traits associated with differential susceptibility to cannabinoid-induced developmental programming. This work was conducted within the same experimental cohort, timeline, and animals as our previous study [31]. These findings further indicate that the effects of PTE in offspring depend on an individual’s stress-coping ability and involve changes in the GI system and gut microbiota composition. This is supported by extensive evidence showing that the ECS regulates intestinal motility [33], barrier function [34], and microbiota homeostasis [34]. In addition, ECS activation has been associated with anti-inflammatory effects [35,36]. Baseline differences in gut microbiota composition and intestinal integrity between Dom and Sub mice further support this premise [29].

In dams, PTE induced microbiota alterations selectively in the Sub phenotype, characterized by enrichment of pro-inflammatory taxa (e.g., *Bilophila*, *Clostridiaceae*) and depletion of genera previously described as short-chain fatty acid (SCFA) producers (Bacteroides, Parabacteroides), whereas Dom dams remained largely resilient.

These findings suggest that the physiological and neuroendocrine milieu of the Sub phenotype may act as a priming factor, lowering the threshold for THC-induced perturbations of the gut–brain–microbiota axis. In this context, the Dom/Sub phenotype represents the pre-existing host background, whereas PTE represents a developmental pharmacological challenge that may interact with this background to produce distinct maternal and offspring microbiome outcomes [37].

The enrichment of Bilophila, a genus previously described as a pathobiont associated with intestinal inflammation and impaired barrier integrity in other models, is consistent with a shift toward a taxonomic profile that has been linked to dysbiosis [38]. Concurrent increases in the Clostridiaceae and Coriobacteriaceae genus are consistent with this compositional pattern. These changes were accompanied by reduced *Parabacteroides* and *Bacteroides* taxa previously reported as SCFA producers [39,40]. Together, these compositional changes indicate a shift toward a microbial profile that has been associated with pro-inflammatory and dysregulated gut environments in other studies [41,42]. Together, these findings suggest that PTE promotes a pro-inflammatory and dysregulated gut environment in Sub dams, potentially amplifying phenotype-dependent differences in offspring responses to PTE.

In contrast, Dom dams appeared relatively protected, potentially due to more efficient stress-regulatory mechanisms that may buffer ECS destabilization induced by exogenous cannabinoids, thereby preserving gut microbiota stability [43]. This stability is critical, as the maternal microbiome serves as the primary source of microbial colonization for the offspring. The ECS is highly active during gut development, regulating intestinal growth and motility by modulation of cholinergic signaling and gut composition [13]. Animal studies indicate that maternal cannabis exposure alters ECS regulatory functions, leading to altered gut morphogenesis [34] and impairing GI motility [44,45]. Moreover, maternal cannabis use has been linked to intrauterine growth restriction (IUGR), which may compromise gut development [46].

Specifically, PTE offspring showed opposing phenotype-dependent effects on gut morphology, reducing gut and colon length in Dom but not Sub mice. As gut and colon lengths were measured at PND 30, these findings are best interpreted as altered postnatal intestinal growth trajectories following prenatal exposure rather than as acute THC-induced shortening of the intestine.

In evaluating offspring intestinal morphometry, it is important to consider systemic growth trajectories. In our previous study, we demonstrated that PTE causes a transient reduction in body weight at PND 30 in Dom offspring but had no effect on Sub offspring body weight [31]. The persistence of GI and microbial shifts in Sub offspring in the absence of weight alterations indicates that PTE exerts line-specific, direct developmental effects on the gut axis independent of generalized growth stunting. This divergence likely reflects differential ECS activity during development and distinct responses to exogenous cannabinoids.

Consistently, PTE increased colonic *CB1R* expression in Dom offspring while reducing *PPARγ* expression in Sub offspring, consistent with phenotype-specific transcriptional changes in genes linked to endocannabinoid signaling and epithelial regulation. Given the role of *CB1R* signaling in gut motility and inflammatory regulation, its upregulation in Dom mice may be associated with the observed GI alterations. In the colon, *CB1R*s are widely expressed in the enteric nervous system, epithelial cells, and immune cell populations, playing a crucial role in modulating motility, secretion, barrier integrity, and appetite regulation [47]. *CB1R* upregulation has been reported in pathological conditions such as inflammatory bowel disease and metabolic syndrome [48,49], where it may exert compensatory anti-inflammatory and anti-motility effects. Consistently, trinitrobenzene sulfonic acid (TNBS)- and Dextran sulfate sodium (DSS)-induced colitis models show that increased *CB1R* expression is associated with reduced inflammation and tissue damage [44,50]. Likewise, modulation of *CB1R* signaling with the antagonist rimonabant (SR141716A) can attenuate intestinal inflammation and restore gut barrier function [44,48], partly through reduced endotoxemia and Toll-like receptor 4 signaling [51].

However, chronic overactivation of the *CB1R* may also contribute to intestinal barrier dysfunction [33] by inhibition of cAMP/PKA signaling, leading to downregulation of tight junction proteins [33], including occludin and zonula occludens-1 (ZO-1) [52,53]. Furthermore, chronic *CB1R* activation in intestinal epithelial cells and enteric neurons can disrupt motility, promote dysbiosis, and induce local hypoxia, collectively fostering a pro-inflammatory microenvironment [54].

Given that *CB1R* upregulation has been associated with intestinal dysfunction in other models, we propose as a hypothesis that the reduced gut length in Dom PTE offspring could relate to altered motility or a pro-inflammatory intestinal environment.

Microbiota analyses revealed bidirectional remodeling of microbial communities. While the Dom offspring showed no significant changes in Shannon diversity following PTE, the Sub offspring displayed a marked reduction compared with controls. Reduced Shannon diversity reflects decreased microbial richness or evenness, potentially compromising ecological stability and host–microbe interactions [55]. However, higher α diversity should not automatically be interpreted as a healthier profile, because the biological meaning of diversity depends on taxonomic composition, host inflammatory state, and functional output [56].

The reduced alpha diversity observed in Sub PTE offspring indicates an alteration in microbial community structure; however, the functional consequences of this reduction cannot be determined from taxonomic diversity alone.

Interestingly, this reduction in alpha diversity occurred alongside improved sociability in Sub PTE offspring, suggesting that lower taxonomic diversity is not necessarily indicative of an adverse behavioral or physiological phenotype. Rather, specific changes in community composition or microbial functional capacity may be more relevant to host phenotypes than overall taxonomic richness or diversity.

Phenotype-dependent shifts in β diversity were associated with behavioral outcomes. Importantly, PTE did not shift Dom and Sub microbiota in the same taxonomic direction. Rather, Dom and Sub offspring exhibited opposite changes in major bacterial groups, including *Firmicutes* and *Bacteroidetes*, while their overall microbial profiles became more similar after exposure. This pattern parallels our previous behavioral findings, in which PTE exerted adverse effects in Dom offspring but beneficial effects in Sub offspring, including enhanced sociability and reduced anxiety-like behavior, thereby diminishing the baseline behavioral separation between the two phenotypes [22]. Thus, the present microbiome data suggest that prenatal THC exposure may partially reduce the original Dom–Sub phenotypic distance through opposite, phenotype-specific gut–brain-axis adaptations rather than through a shared unidirectional response. The microbial community of both strains overlapped upon PTE, while the Sub offspring shifted toward a Dom-like cluster, aligning with improved sociability and reduced anxiety. Conversely, Dom PTE offspring shifted toward a Sub-like profile, paralleling increased anxiety and reduced resilience. This bidirectional re-clustering suggests that PTE was associated with phenotype-dependent shifts in gut microbiota composition that paralleled baseline stress-coping strategy, consistent with a possible interplay between host behavior and microbiota in shaping long-term neurobehavioral outcomes.

At the taxonomic level, PTE induced opposing changes in key microbial genera associated with stress [56,57,58,59], inflammation [56,57,60,61,62], and mood regulation [59,63,64,65].

Increased abundance of *Lachnospiraceae*-related taxa in Dom PTE offspring correlated with the reported anxiety-like behavior [31]. Although *Lachnospiraceae* have been reported to contribute to butyrate production and gut homeostasis in other studies [66,67], their overrepresentation has also been linked to mucosal damage and inflammation [56].

Similarly, maternal immune activation (MIA) models show increased *Lachnospiraceae* abundance alongside behavioral impairments, including reduced sociability and increased anxiety [63]. In agreement with these reports, Dom PTE offspring exhibited elevated levels of *Lachnospiraceae* genera (e.g., *Lachnoclostridium*, NK4B4, *Acetatifactor*, UCG-001), paralleling anxiety-like behavior [31]. In contrast, these taxa (*Lachnoclostridium*; *Lachnospiraceae* FCS020, NK4A136, UCG-006, *Roseburia*, *Acetatifactor,* NK4B4, and *Eubacterium xylanophilum*) were reduced in Sub PTE offspring, corresponding with improved sociability and reduced anxiety-like behavior [31]. These findings suggest that *Lachnospiraceae* abundance is associated with stress vulnerability and social phenotype. Given the role of *Lachnospiraceae* in SCFA production and GBA regulation, their different abundance across Dom and Sub lineages may reflect differential metabolic and neuroimmune adaptations associated with phenotype-specific behavioral outcomes. Overall, these findings suggest that social dominance interacts with PTE and corresponds to shifts in *Lachnospiraceae* dynamics, potentially influencing susceptibility to neurodevelopmental disturbances.

Additional taxa affected by PTE included *Mucispirillum* and *Ligilactobacillus.* Sub PTE offspring exhibited reduced *Mucispirillum* abundance, whereas increased levels of this genus have been linked to anxiety- and depressive-like behaviors and reduced body weight in chronic social defeat models [64], suggesting an association with stress vulnerability. In contrast, PTE increased *Ligilactobacillus* abundance in Sub offspring while reducing it in Dom offspring. This genus has been associated with stress regulation, sociability, and metabolic outcomes [58,60,65]. Experimental studies show that *Ligilactobacillus* enhances antioxidant capacity, supports body weight gain, and improves gut microbiota composition [58], while also preserving colon length and reducing inflammation in colitis models [60]. In an LPS-induced depression model, increased *Ligilactobacillus* abundance was linked to restored hippocampal serotonin and BDNF levels, reduced inflammation, and improved behavior, with similar effects observed following direct supplementation [65].

These prior reports raise the possibility that the phenotype-specific shift in *Ligilactobacillus* abundance we observed is relevant to the behavioral differences between Dom and Sub PTE offspring.

In addition, the gut microbiota profile of Dom PTE offspring showed reduced abundance of *Rikenella* and *Muribaculum* compared with Dom Veh. *Rikenella*, previously enriched in female offspring following MIA, has been associated with anti-inflammatory and protective effects that may support resilience in social behavior [61]. Similarly, decreased *Muribaculum* abundance in chronically stressed mice has been linked to shorter colon lengths, reduced body weight, decreased sociability, and increased anxiety [59].

Taken together, these findings suggest that prenatal THC exposure does not exert a uniform effect on the gut–brain axis, but instead, interacts with the pre-existing Dom/Sub phenotype to produce opposite microbiota, intestinal, and behavioral outcomes that ultimately reduce the original phenotypic distance between stress-resilient and stress-vulnerable offspring.

Several limitations should be considered when interpreting these findings. First, the study is observational with respect to microbiota–host relationships: no germ-free colonization, microbiota transplantation, or *CB1R* pharmacological manipulation was performed, so the associations reported here between microbial taxa, colonic gene expression, and previously described behavioral phenotypes cannot be interpreted as causal or mechanistic. Second, the sample size limits statistical power, particularly for detecting phenotype × treatment interactions and for fully modeling litter as a random effect; we mitigated this by capping pups per litter and by confirming the absence of dam-dependent effects across all offspring outcomes, but a larger cohort would allow more robust hierarchical modeling. Third, only male offspring were used, and given known sex differences in endocannabinoid signaling, stress reactivity, and gut microbiota composition, these findings cannot be generalized to females. Fourth, 16S rRNA sequencing resolves taxonomic composition but not microbial function or metabolite output; metagenomic or metabolomic approaches would be needed to test the functional hypotheses raised here. Finally, molecular and microbiome endpoints were assessed at a single postnatal timepoint (PND 30), so the persistence or trajectory of these changes beyond this age remains untested. These, and multiple other questions, require future investigation.

## 4. Materials and Methods

### 4.1. Animals

In this study, mice with strong features of dominance and submissiveness were used. Dominant (Dom) and Submissive (Sub) mice were produced using the outbred Sabra strain [68] (Envigo, Ness Ziona, Israel) based on a selective breeding approach and Dominance–Submissiveness Relationship (DSR) behavioral paradigm [69], a food competition test assessing social interactions within fixed pairs of mice [24,26,69]. In this test, more than 99% of selectively bred Dom and Sub mice consistently develop robust and stable dominant or submissive relationships. Generation 54 was used in this study (in-house breeding). After weaning, mice were housed in a colony room (12:12 L:D cycle with lights on 07:00–19:00, 25 ± 2 °C, 55 ± 5% humidity) in groups of five per cage and provided with standard laboratory chow and water ad libitum.

To evaluate prenatal exposure while accounting for potential litter dynamics and avoiding pseudoreplication, pregnant dams were treated as the primary experimental unit. A total of 32 dams were assigned across four treatment groups: Dom Vehicle (*n* = 8), Dom PTE (*n* = 8), Sub Vehicle (*n* = 8), and Sub PTE (*n* = 8). At delivery, litters were culled to eight pups per dam.

At weaning (PND 24), offspring were separated into single-sex housing to prevent unplanned breeding. Only male offspring were retained for experimental use. To limit litter-related confounding, no more than 3 pups per litter were allocated to any single downstream analytical group (morphology, gene expression, or microbiome profiling), and all primary offspring analyses (Section 4.6) account for dam/litter of origin statistically rather than relying on this cap alone. Dam stool microbiota was analyzed in a subset of dams per group (*n* = 4–5/group) due to insufficient read depth/DNA yield in excluded samples. Sample size and distribution are shown in Table S1. Gut/colon length measurements and qRT-PCR data acquisition were performed by laboratory personnel who were not informed of the specific treatment or phenotype group assigned to each sample, and samples were processed using coded identifiers rather than group labels. Microbiome bioinformatic analyses (QIIME2, ANCOM-BC) were performed using standardized, automated pipelines applied uniformly across all samples.

**Figure 5:**
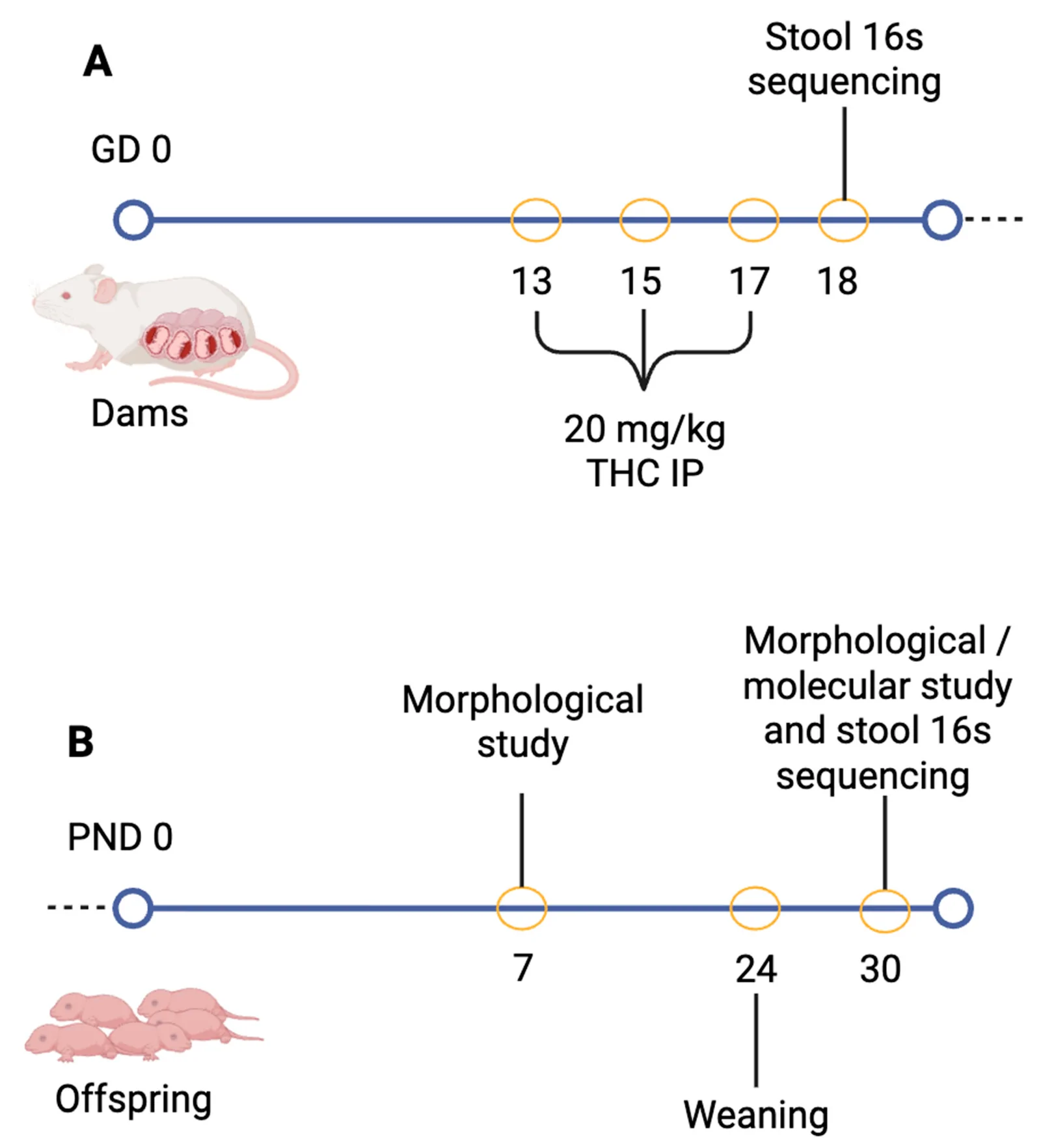
Study design. (**A**) Dams: 20 mg/kg THC was administered (i.p.) to pregnant mice at GD 13, 15, and 17. Stools were collected from both dams (GD 18). (**B**) Offspring: Gut and colon tissues were collected for morphometric analysis at PND 7 and PND 30; colonic tissue for molecular analyses was collected at PND 30 only. Stools were collected from offspring at PND 30. This figure was created with BioRender.com.

Mice were euthanized via CO_2_ inhalation. All experimental procedures were conducted in accordance with NIH/USDA and ARRIVE guidelines and were approved by the Ariel University Institutional Animal Care and Use Committee (permission numbers: AU-IL-2112-105-3 (approval date 7 December 2021) and AU-IL-2112-108-3 (approval date 30 December 2021). The overall study design is illustrated in Figure 5.

### 4.2. ∆^9^-Tetrahydrocannabinol (THC) Preparation

To produce ∆^9^-THC from cannabidiol (CBD), 3.14 g of cannabidiol was dissolved in 100 mL dry dichloromethane, where 400 mg pre-heated MgSO_4_ was dispersed, 25 μL of BF_3_ diethyl etherate was added, and the reaction was mixed at −15 °C for 1.5 h in an N_2_ atmosphere. Then, the reaction was quenched with 20 mL ice-cold NaHCO_3_-saturated solution, the phases were separated, and the aqueous phase was extracted with two portions of 30 mL dichloromethane (DCM). The combined organic phase was washed to neutrality with a saturated NaCl solution, dried over MgSO_4,_ and evaporated. The yield of ∆^9^-THC was 82.3%, and the rest of the material remained unreacted CBD. The purity of THC used for treatment was >97%, as confirmed using LC-MS.

### 4.3. THC Administration

To test the effects of prenatal THC exposure in Dom and Sub offspring mice, two-month-old females were mated with males of the corresponding behavioral phenotype. The presence of a vaginal plug was designated as gestational day (GD) 0.

On GD 10, pregnant females were single-housed and randomly assigned to vehicle or THC treatment using a randomized allocation sequence generated prior to the start of dosing, stratified by behavioral phenotype (Dom/Sub) to ensure balanced group sizes. Dom and Sub dams were i.p. injected with 200 μL vehicle (1:1:18 ethanol: Tween-80: saline) (*n* = 8/group) or synthetic THC (20 mg/kg, *n* = 8/group) on GD 13, 15, and 17 between 09:00 and 12:00 h. The THC dose was selected based on our previous study [25]. The GD13, GD15, and GD17 exposure windows were chosen to match our previous PTE protocol in Dom/Sub mice and to target a developmental period during which the fetal ECS participates in neurodevelopmental and gastrointestinal maturation [22,33].

### 4.4. Molecular Studies

Colonic tissue was collected at PND 30 (as shown in Figure 5) and stored at −80 °C until use. Total RNA was extracted using the RNeasy Micro Kit (Qiagen Cat. No./ID: 74104). Reverse transcription was performed using the GoScript™ Reverse Transcription System (Promega, A3801, Madison, WI, USA) according to the manufacturer’s instructions. Quantitative real-time PCR (qRT-PCR) was carried out using the SYBR™ Green PCR Master Mix (Applied Biosystems, Waltham, MA, USA, Cat. No. 4309155) on a QuantStudio Real-Time PCR System. Primer sequences are provided in Table 1.

Primer efficiency was determined for each gene using standard curves generated from serial cDNA dilutions; all efficiencies fell within 91.5–96.9%. *HPRT* Ct values did not differ significantly by phenotype, treatment, or their interaction (two-way ANOVA, *p* > 0.05 for all), confirming its suitability as a reference gene across experimental groups. Melt curve analysis showed a single dissociation peak for each primer pair, confirming amplification specificity.

### 4.5. Gut Microbiota Analysis

#### 4.5.1. Stool DNA Extraction and 16S rRNA Amplicon Sequencing

Fresh stools were collected and stored at −80 °C. Genomic DNA was extracted using the QIAamp DNA Stool Mini Kit (QIAGEN GmbH, Hilden, Germany; Cat. No. 51504). Extraction was performed by Syntezza Bioscience (Jerusalem, Israel). Purified DNA samples were stored at −20 °C until further processing.

16S rRNA amplicon library preparation and sequencing were performed by Syntezza Bioscience (Israel). Briefly, the V4 hypervariable region of the 16S rRNA gene was PCR-amplified using region-specific primers (F515 (5′-CACGGTCGKCGGCGCCATT-3′) and R806 (5′-GGACTACHVGGGTWTCTAAT-3′)) incorporating unique per-sample barcode sequences. Amplicon fragments were purified, quantified, and constructed into sequencing libraries. Libraries were sequenced on an Illumina platform using paired-end sequencing to generate FASTQ reads, which were subsequently processed using the QIIME2 pipeline as described below.

#### 4.5.2. Taxonomic Analyses of 16S rRNA Gene Sequencing

FASTQ data were processed and analyzed using the QIIME2 pipeline (version 2023.5; QIIME 2, Caporaso Lab, Northern Arizona University, Flagstaff, AZ, USA) [70]. Paired-end sequences were demultiplexed with the q2-demux plugin. Reads were denoised and clustered using DADA2 (q2-dada2) to improve taxonomic resolution [71]. Prior to quality filtering, raw read counts ranged from 862,023 to 1,793,894 reads/sample for dams and from 39,053 to 64,427 reads/sample for offspring. After denoising and chimera removal (DADA2), non-chimeric read counts ranged from 147,280 to 277,037 reads/sample for dams (*n* = 19) and from 31,423 to 52,359 reads/sample for offspring (*n* = 20); no samples were excluded during quality control.

Taxonomic classification was performed with the q2-feature-classifier, and feature sequences were aligned to the Greengenes database (v.12.10) with 99% confidence [72]. The feature table was filtered using q2-feature table to remove contamination. Features annotated as mitochondria or chloroplasts were excluded. Additionally, features found in ≤10% of samples per group or with total frequencies < 0.001% were removed. Samples were rarefied to an even sequencing depth before diversity analyses, using the lowest non-chimeric read count within each dataset as the rarefaction threshold: 10,000 reads/sample for dams and 31,423 reads/sample for offspring. α diversity was calculated using observed species richness and the Shannon diversity index with the plotRichness function in phyloseq (v1.50.0) [73]. Statistical comparisons between experimental groups were performed using pairwise Wilcoxon rank-sum tests.

β diversity was assessed by principal coordinates analysis (PCoA) based on Bray–Curtis dissimilarity using the ordinate function in phyloseq. Significance was assessed by permutational multivariate analysis of variance (PERMANOVA) [74]. PERMANOVA models included phenotype, PTE, and phenotype × PTE interaction terms, and results are reported as *p* values for each term. Taxonomic composition was analyzed at the phylum and genus levels using TaxGlom. Relative abundance was calculated as a percentage of total reads per sample using transformed sample counts. Taxa were filtered by median relative abundance per experimental condition: phyla with >1% and genera with >2.5% median abundance were displayed individually, while rare taxa were grouped as “<1%” or “<2.5%”, respectively. Stacked bar charts were generated with ggplot2 using colors from the Paired palette (RColorBrewer (version 1.1-3; Erich Neuwirth, University of Vienna, Vienna, Austria)). Summary tables reporting mean ± standard deviation for each taxon per group were produced using dplyr. All analyses were performed in R (v4.4.1) [75,76].

#### 4.5.3. ANCOM-BC Analysis

Differential abundance testing was performed using the Analysis of Compositions of Microbiomes with Bias Correction (ANCOM-BC) framework [77], implemented in the ANCOMBC R package. Analyses were conducted at the phylum and genus taxonomic levels. For each pairwise comparison, taxa were agglomerated using the TaxGlom function at the specified taxonomic level, and the ANCOM-BC function was applied with experimental condition as the fixed effect (formula = “Condition”). Multiple testing correction was applied using the Benjamini–Hochberg false discovery rate (FDR) method [78]. Taxa with FDR-adjusted q values < 0.05 were considered differentially abundant. Log fold change (LFC) values represent the bias-corrected difference in log-transformed relative abundance between groups. Results were visualized in lollipop plots; individual abundance boxplots were generated for each significantly differentially abundant taxon using ggplot2 (v3.5.1) [75,77].

### 4.6. Statistical Analysis

Data were analyzed using a 2 × 2 factorial design with phenotype (Dom vs. Sub) and treatment (Vehicle vs. PTE) as fixed factors. Comparative analysis between two groups (Dom vs. Sub) was performed using Student’s *t*-test. For multiple group comparisons involving factors such as type and treatment, two-way ANOVA was employed. Sidak’s correction was used for comparisons between fixed pairs within groups. Lack of dam-dependent effects were verified by two-way ANOVA. All data are expressed as mean ± SEM. The *t*-test and two-way ANOVA outputs, including F statistics, degrees of freedom, *p* values, and sample sizes, are reported in the text and figure legends. Statistical significance for ANOVA comparisons is indicated as * for *p* < 0.05, ** for *p* < 0.01, *** for *p* < 0.001, and **** for *p* < 0.0001. Significance for Student’s *t*-test comparisons is indicated as ^#^ for *p* < 0.05, ^##^ for *p* < 0.01, ^###^ for *p* < 0.001, and ^####^ for *p* < 0.0001.

## 5. Conclusions

Our findings demonstrate that prenatal THC exposure (PTE) produced phenotype-dependent alterations in intestinal morphology, selected colonic transcripts, and gut microbiota composition in Dom and Sub offspring. Dom PTE offspring exhibited adverse outcomes, including GI dysmorphology by reducing gut and colon lengths, gut microbiota dysbiosis, and elevated anxiety-like behavior [31]. In contrast, stress-vulnerable Sub offspring exhibited a distinct behavioral profile characterized by reduced anxiety-like behavior despite a concomitant decrease in microbial diversity. This bidirectional response supports the concept that established stress-resilient and stress-vulnerable phenotypes differ in their gut–brain-axis response to prenatal cannabinoid exposure. Together with previous evidence that Dom and Sub mice differ in baseline microbiota and behavioral responses to PTE, the present findings suggest that microbiome-related mechanisms may be associated with phenotype-dependent developmental outcomes following prenatal THC exposure [22,30]. Furthermore, prenatal environmental triggers, such as cannabis, may intervene in this process in both stress-resistant and stress-vulnerable individuals with distinct outcomes. Understanding this interindividual variability may help guide phenotype-specific interventions for the prevention of pathologies during early stages of prenatal development.

## Figures and Tables

**Table 1:** Primer sequences list used in the study.

*Gene*	Forward	Reverse
*CB1R*	GTCGATCTTAGACGGCCTTGC	TTGAGCCCACGTAGAGGAGGT
*FAAH*	CAGTATGTGTCCTCGGTCAGC	CCACGTCCAAGTTCATTGCC
*CLDN4*	AACTGCATGGAGGACGAGAC	GGGTTGTAGAAGTCGCGGAT
*CLDN7*	TGTCTTGTGGAGGGCTTGAG	TCCATCCAGAGCCCCTTGTA
*GPR43*	GGGAGAGGATCTGAGGAGCA	CACCTGCCAGAACTCCTTGG
*PPARγ*	GCCAGTTTCGATCCGTAGAA	ATTCCTTGGCCCTCTGAGAT
*HPRT*	GCTTGCTGGTGAAAAGGACC	TGCAGATTCAACTTGCGCTC

## Data Availability

The original contributions presented in this study are included in the article/supplementary material. Further inquiries can be directed to the corresponding authors.

## Supplementary Materials

The following supporting information can be downloaded at: https://www.mdpi.com/article/10.3390/ph19091463/s1. Supplementary Figure S1: Gut and colon length in neonatal (PND 7) Dom and Sub offspring following prenatal THC exposure; Supplementary Figure S2: Colonic gene expression of gut permeability and endocannabinoid-related markers in offspring of Dom and Sub following prenatal THC exposure at PND 30; Table S1: Sample size and distribution; Table S2: Beta diversity analysis; Table S3: Microbiota abundance analysis sheet.

## Abbreviations

The following abbreviations are used in this manuscript:

THC	Δ^9^-tetrahydrocannabinol
PTE	Prenatal THC exposure
PCE	Prenatal cannabis exposure
ECS	Endocannabinoid system
GBA	Gut–brain axis
GI	Gastrointestinal
*CB1R*	Cannabinoid receptor 1
*PPARγ*	Peroxisome proliferator-activated receptor gamma
