# Supplementary material for: Prenatal THC Exposure Differentially Alters Gut Physiology and Microbiota in Stress-Resilient and Vulnerable Mice
Source: Pharmaceuticals (Basel). 2026 Sep 15;19(9):1463. doi: 10.3390/ph19091463 (PMC13610397; doi:10.3390/ph19091463)
Supplement: Supplementary file 1 [file pharmaceuticals-19-01463-s001.zip › pharmaceuticals-4501764-supplementary.pdf]

Table S1: Sample size and distribution

Analysis	Mice	Dams Contributing/group	n analyzed/group
**Dam stool microbiota (Fig. 1)**	Dams	4-5	4-5
**Offspring gut/colon length (Fig. 2A,B)**	Male Offspring	2-3	6-8
**Offspring colonic mRNA (Fig. 2C,D)**	Male Offspring	2	4-5
**Offspring stool microbiota (Fig. 3)**	Male Offspring	2	4-5

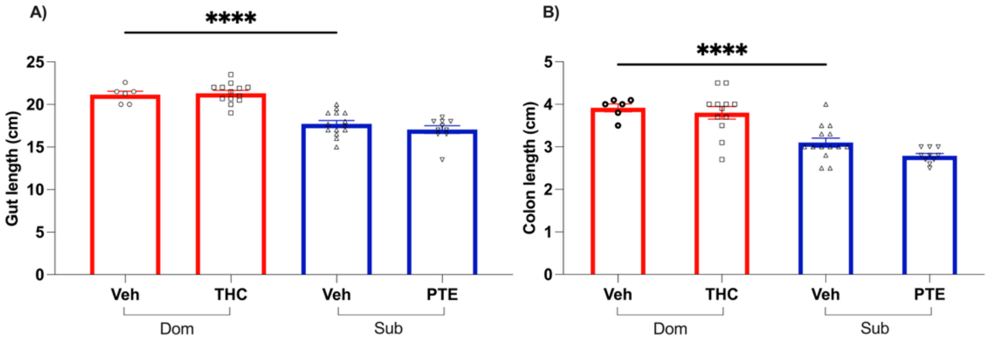

Supplementary Figure 1. Gut and colon length in neonatal (PND 7) Dom and Sub offspring following prenatal THC exposure. Total gut length (A) and colon length (B) were measured in the Dom and Sub offspring at PND 7. Sub Veh offspring exhibited significantly shorter gut and colon lengths compared to Dom Veh offspring. Data are mean ± SEM (gut/colon: n = 6 – 10 per group). Statistical analysis was performed by One-way ANOVA (Tukey correction); **** at $p < 0.0001$.

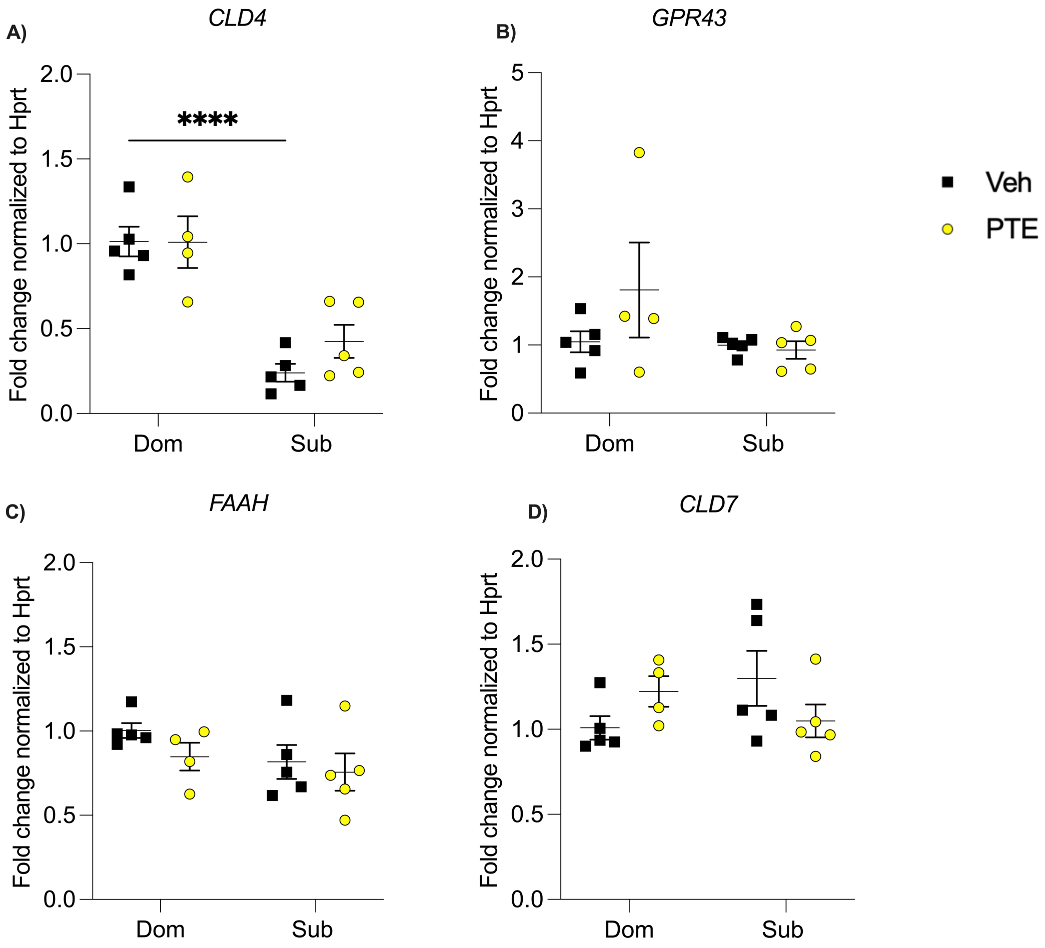

Supplementary Figure 2. Colonic gene expression of gut permeability and endocannabinoid-related markers in offspring of Dom and Sub following prenatal THC exposure at PND 30. Fold change in mRNA expression of **(A)** *CLD4*, **(B)** *GPR43*, **(C)** *FAAH*, and **(D)** *CLD7*, normalized to *Hprt*, in colonic tissue of Dom and Sub offspring. Data are mean ± SEM (mRNA: n=4-5/group). Statistical analysis was performed by two-way ANOVA (Sidak correction); **** at $p < 0.0001$.
